# Kinetics of Low Temperature Polyester Dyeing with High Molecular Weight Disperse Dyes by Solvent Microemulsion and AgroSourced Auxiliaries

**DOI:** 10.3390/polym10020200

**Published:** 2018-02-16

**Authors:** Shahram Radei, F. Javier Carrión-Fité, Mònica Ardanuy, José María Canal

**Affiliations:** Secció Enginyeria Tèxtil, Departament de Ciència dels Materials i Enginyeria Metal.lúrgica, Universitat Politècnica de Catalunya, C/Colom 11, 08222 Terrassa, Spain; radeishahram@yahoo.com (S.R.); javier.carrion@upc.edu (F.J.C.-F.); jose.maria.canal@upc.edu (J.M.C.)

**Keywords:** polyester dyeing, microemulsion, *o*-vanillin, coumarin, *n*-butyl acetate, kinetics

## Abstract

This work focused on the evaluation of the kinetics of dyeing polyester fabrics with high molecular weight disperse dyes, at low temperature by solvent microemulsion. This study also compared the effect of two non-toxic agro-sourced auxiliaries (*o*-vanillin and coumarin) using a non-toxic organic solvent. A dyeing bath consisting of a micro-emulsion system involving a small proportion of *n*-butyl acetate was used, and the kinetics of dyeing were analysed at four temperatures (83, 90, 95 and 100 °C). Moreover, the dyeing rate constants, correlation coefficient and activation energies were proposed for this system. It was found that *o*-vanillin yielded higher dye absorption levels than coumarin, leading to exhaustions of 88% and 87% for Disperse Red 167 and Disperse Blue 79, respectively. *K*/*S* values of dyed polyester were also found to be higher for dye baths containing *o*-vanillin with respect to the ones with coumarin. In terms of hot pressing fastness and wash fastness, generally no adverse influence on fastness properties was reported, while *o*-vanillin showed slightly better results compared to coumarin.

## 1. Introduction

Polyester (Polyethylene terephthalate) (PES) fibres are the most used fibres in the global apparel industry, and therefore are a prominent topic of research. The hydrophobic nature PES fibres, combined with a glass transition temperature (*T*_g_) of around 80 °C, make their dyeing process challenging, since it is high-energy and consumes a significant amount of water [1]. The most common dyestuffs used to dye PES fibres are disperse dyes. These dyes are almost insoluble in water, making it necessary to use them in combination with dispersing agents and carriers, if the dyeing processes are performed in water under 100 °C [2,3]. The carriers, which have smaller size than the dyes, are capable of penetrating the amorphous regions of fibres, like polyester, and opening the macromolecular structure of the polymer at temperatures higher than the *T*_g_. However, the nature and high molecular weight of the organic compounds used generally as carriers involves problems of non-biodegradability and toxicity. These agents can also partially plasticize polyester fibres, decreasing their glass transition temperature [4,5].

Dyeing and avoiding the use of carriers in the conventional industrial process is only possible at higher temperatures, such as 130–135 °C, to produce a sufficient absorption level. Although this system is the most used process in the world, it requires costly machinery in relation to the material processed, and a considerable amount of energy to heat and maintain the dye bath at the operating temperature [6]. 

Recent studies have attempted to look for alternative processes for polyester dyeing with non-hazardous and more environmentally friendly compounds that also require less energy in the overall dyeing process. With the aim of avoiding negative effects of the carriers, Harifi and Montazer [7] studied the effect of nano-TiO_2_ on the dyeing process of PES with disperse dyes. They found that the colour strength of the PES fabric increased by the effect of the pre-treatment, consisting of an immersion in aqueous dispersions of nano-TiO_2_ particles (0.25%–2% (*w*/*v*)). This method was free from the aforementioned disadvantages of carriers, but its industrial application is costly and very difficult to perform [7]. 

Choi et al. [8] compared the dyeing behaviour of PES fabrics with Disperse Blue C.I. 56 dye, and used two environmentally friendly cationic gemini surfactants as auxiliaries, namely propanediyl-α,ω-bis(dimethyldodecylammoniumbromide) (DC3-12) and hexanediyl-α,ω-bis (dimethyldodecylammoniumbromide) (DC6-12) or a conventional surfactant (dodecyltrimethylammonium bromide) (C12C1NBr). The study proved that colour strength with gemini surfactants was higher than achieved with the conventional one. They found also that increasing the concentration of gemini surfactants led to a decrease of the dye hydrophobicity above the critical micelle concentration (CMC). Moreover, it was found that dyeing with gemini surfactants had benefits such as controlling dyeing kinetics and improving dye absorption by the PES fabric [9].

On the other hand, several studies propose dyeing processes with more environmentally friendly carriers at temperatures lower than 100 °C. Pasquet et al. [10], used a high concentration of *o*- and *p*-vanillin as carriers for low-temperature dyeing of polyester fabrics (90 °C), with low and high molecular weight disperse dyes in the presence of ethanol as a co-solvent [10]. This study proved that *o*- and *p*-vanillin can be a substitute for conventional carriers. Additionally, PES fabric dyed with these carriers presented acceptable absorption and fastness. Furthermore, Pasquet et al. [10] analysed the toxicity of *o*- and *p*-vanillin with the USEtox system, confirming that these carriers are not toxic. Carrión-Fite proposed a low-temperature dyeing process in the presence of a micro-emulsion obtained by ultrasonic agitation, composed of a low proportion an alkyl halogen solvent and a phosphoglyceride emulsifier [11]. In this sense, it was proved that ultra-sonic cavitation improves the dyeing rate constant, and the dye absorbed by the fibre [12,13].

More recently, Carrión-Fite and Radei [14] proposed the use of more environmentally friendly dyeing auxiliaries based on *o*-vanillin or coumarin in an *n*-butylacetate co-solvent (an organic non-toxic solvent) for low molecular weight disperse dyes (C.I. Disperse Blue 56 and C.I. Disperse Red 73). The study confirmed that *o*-vanillin and coumarin had a positive and significant influence on dyeing behaviour of polyester in this system. At a temperature of 95 °C, the absorption levels of the fibres after 120 min were excellent: the content of Disperse Blue 56 dye absorbed by the PES in the presence of *o*-vanillin or coumarin was 97% and 96%, respectively. In the case of Disperse Red 73, the amount absorbed was 81% and 82%, respectively, for of *o*-vanillin or coumarin. Moreover, wash fastness was similar to the conventional process of dyeing polyester [14,15]. However, it was necessary to study more in depth the effect of the carriers by analysing the thermodynamic parameters and heat fastness, among others, to establish this new methodology. Moreover, another limitation of this study was that only low molecular weight disperse dyes were analysed. 

In this paper, we investigated the kinetics of dyeing PES fabrics with two high molecular weight disperse dyes (Disperse Red 167 and Disperse Blue 79), in a micro-emulsion system involving a small proportion of a non-toxic organic solvent (*n*-butyl acetate), and two potential auxiliaries (*o*-vanillin and coumarin). Moreover, the activation energies were determined for this system. 

## 2. Materials and Methods 

### 2.1. Chemicals

The fabric used was a PES 100% woven standard polyester, Type 30 A, from Testgewebe GmbH (Brüggen, Germany) (ISO 105-F10). The high molecular weight disperse dyes used were Rubi Foron S-2GFL (C.I Disperse Red 167) and Navy Blue Foron S-BRL (Disperse Blue 79). All dyes were supplied by Clariant (Prattlen, Switzerland). Coumarin (99% purity) was supplied by Acros (NJ, USA) and *o*-vanillin (99% purity) was supplied by Acros (NJ, USA). Both carriers use agro-sourced alternative compounds with additional properties, such as antimicrobial, antioxidant, biodegradability and antimutagenic. Also, these carriers are hydrophobic with no toxicity, and we expect them to have better influence on polyester dyeing [10,16]. The chemical structure of these chemicals is presented in Table 1. 

Pure *n*-butyl acetate (*M*_w_ = 116.16 g·mol^–1^), supplied by Panreac (Barcelona, Spain), was used as the co-solvent. *N*,*N*-Dimethylformamide (*M*_w_ = 73.10 g·mol^–1^), 99.8% purity and supplied by Panreac (Barcelona, Spain), was used as the solvent to extract the dyes from the PES fabrics. Hostapal non-ionic surfactant detergent, supplied by Archroma GmbH (Sulzbach, Germany), was used as for pre-dyeing washing. QP-grade 85% pure sodium dithionite (*M_w_* = 174.11 g·mol^–1^) and analytical-grade sodium hydroxide, both supplied by Panreac, were used as a post-dyeing washing reductant.

### 2.2. Experimental Methods

#### 2.2.1. Dyeing Procedure

**Figure 1:**
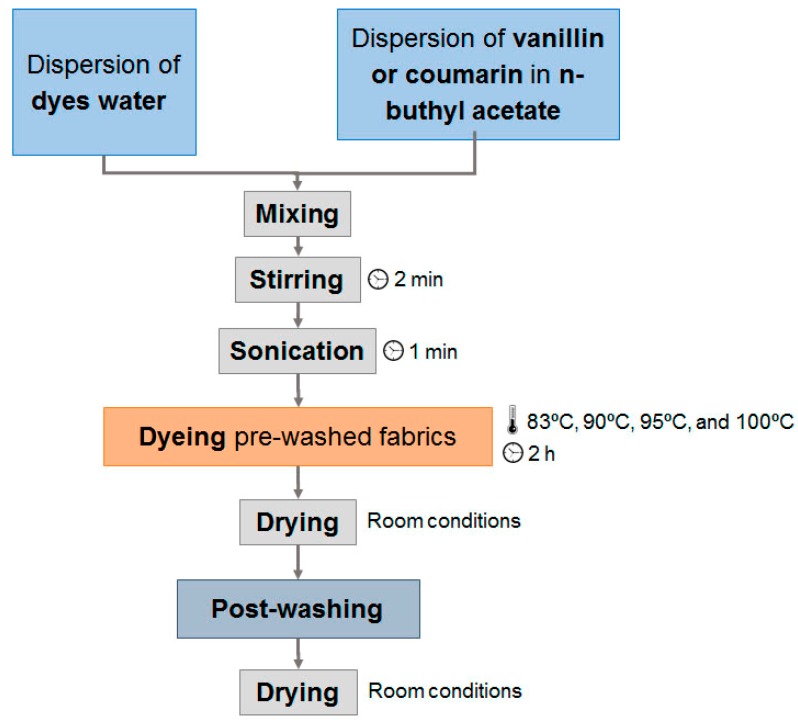
Experimental dyeing process used for this study.

Figure 1 shows the dyeing process for the proposed methodology.

PES fabrics were prepared for dyeing by cutting 16 cm in warp and 11 cm in weft, and weighing 3.0 g. Fabric pre-washing was done by using Hostapal detergent at a 0.5 g·L^–1^ concentration at 40 °C for 30 min.

The dye bath was prepared by dissolving carriers in *n*-buthylacetate and then adding the dye solution. The mixture was stirred for 2 min followed by sonication for 1 min. The bath ratio for each dyeing operation was set to 1:60. The quantity of dyes and chemicals used were 2% o.w.f. of disperse dye, 4% o.w.f. of auxiliaries and 3 ml of *n*-buthylacetate, for a total amount of 180 mL of dye bath. The dyeing process was carried out in a Linitest dyeing apparatus, furnished with 300 mL sealed cans that were purchased from Atlas MTT GmbH (Linsengerich, Germany). The dyeing temperatures were 83, 90, 95 and 100 °C for a fixed time of 120 min. One-half of all tests used *o*-vanillin and the other half coumarin as auxiliaries.

After the dyeing process, the samples were rinsed with water two times and dried at ambient conditions. Post washing was performed in a reductive medium to remove all dye absorbed onto the fibre surfaces. The reductive medium consisted on a mixture of 0.5 g/L sodium hydroxide and 2 g/L sodium hydrosulphite in distilled water (bath ratio 1:50). This process was followed by washing at 50 °C for 30 min, rinsing three times with water and drying at room temperature for 24 h. 

#### 2.2.2. Colour Fastness

Wash fastness of the samples was tested according to the UNE-EN ISO 105-C06 method. The samples were washed with IEC 60456 Base Detergent Type A at 40 °C for 30 min, keeping liquor to material ratio as 1:50. The colour fastness to hot pressing was tested according to the UNE-EN ISO 105-X11 method. 

#### 2.2.3. Dye Absorption

The amount of dye absorbed by the PES was quantified by spectrophotometric analysis after dye extraction with *N*,*N*-dimethylformamide (DMF). The absorbance at the maximum wavelength in the visible spectrum is a linear function of the concentration of dye dispersed in DMF following the Beer–Lambert law. The maximum absorbance was determined for each dye at different concentrations, obtaining linear regressions with R values ranging from 0.9758 to 0.9845. A model M 330 UV–Visible spectrophotometer was used that was purchased from Camspec (Leeds, UK).

#### 2.2.4. Particle Size Analysis

To evaluate the homogeneity of the dye baths, particle size measurements were performed using a Zetasizer nano series device from Malvern (Malvern, UK) at 90° with respect to the incident laser beam at 25 °C [17].

#### 2.2.5. Determination of Exhaustion 

The exhaustion was determined using Equation (1):

(1) $$Exahustion\;\left(\%\right)=\frac{C_{0}-C_{s}}{C_{0}}\times 100$$

where *C*_0_ is initial concentration of dye and *C_s_* is final concentration of dye.

#### 2.2.6. Determination of Colour Strength 

The colour strength, expressed as *K*/*S* value, was determined from the spectral diffuse reflectance using a spectrophotometer (Gretag Macbeth 7000). Reflectance values of the samples (*R*) were used to calculate *K*/*S* based on Kubelka-Munk, as shown in Equation (2) [18]:

(2) $$\frac{K}{S}=\frac{{\left(1-R\right)}^{2}}{2R}.$$

where *R* is the % Reflectance/100 of coloured samples at λ_max_, and *K* and *S* are the absorption and scattering coefficients, respectively.

## 3. Results and Discussion

### 3.1. Emulsion Particle Size

Before dyeing, the particle size of the dye bath emulsions was determined and compared with the bath in the presence of high molecular weight disperse dyes without any auxiliaries. The average values are presented in Table 2.

According to the table, it can be concluded that all dye-baths were in general homogenous: values obtained were acceptable in this micro-emulsion system. The average for the particle size (200 nm) and the mean of polydispersity (0.2) presented standard values for the dyeing process. Particle sizes of dye-bath in a presence of coumarin showed better results. Indeed, presence of this auxiliary successfully reduced the particle size. Nonetheless, when *o*-vanillin was used, no significant changes in particle sizes were found with respect to the bath without auxiliaries. 

### 3.2. Dyeing Kinetics and Rate Constant

**Figure 2:**
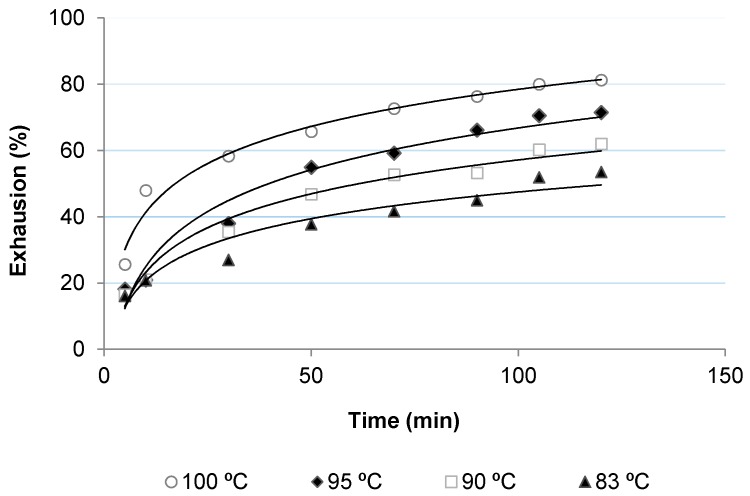
Dyeing kinetics for polyester (polyethylene terephthalate) (PES) fabric dyed with Disperse Red 167 and Coumarin.

**Figure 3:**
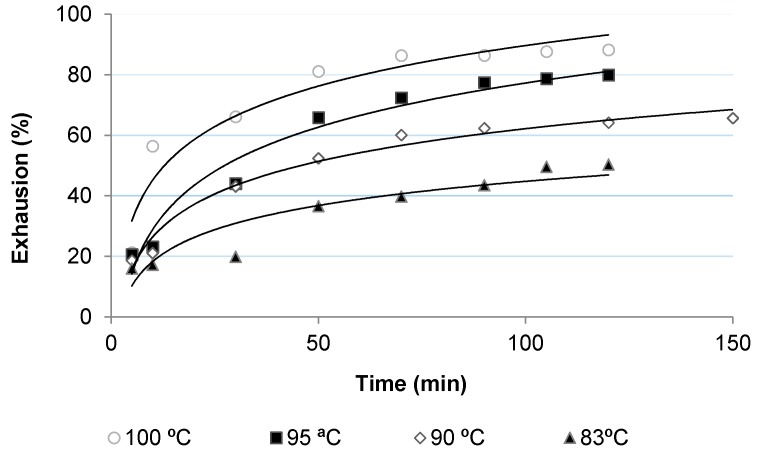
Dyeing kinetics for PES fabric dyed with Disperse Red 167 and *o*-vanillin.

**Figure 4:**
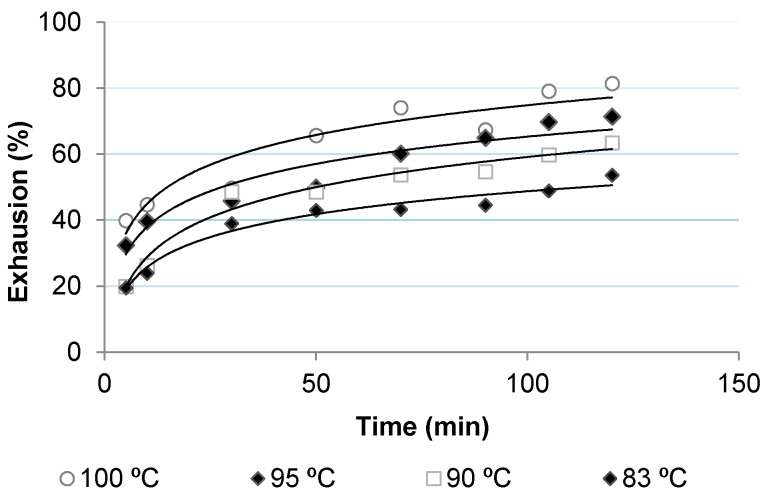
Dyeing kinetics for PES fabric dyed with Disperse Blue 79 and Coumarin.

**Figure 5:**
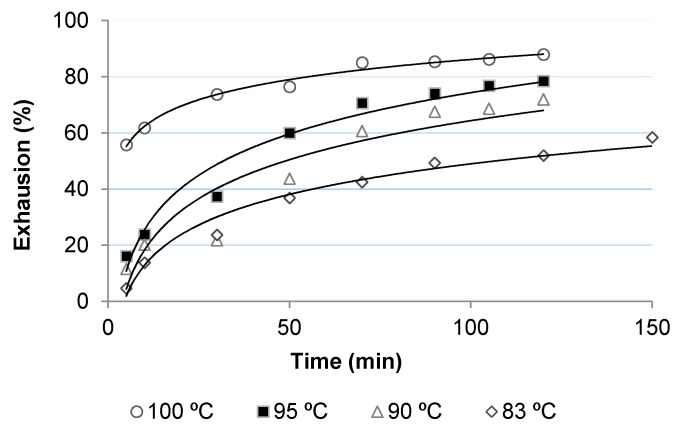
Dyeing kinetics for PES fabric dyed with Disperse Blue 79 and *o*-vanillin.

Figure 2, Figure 3, Figure 4 and Figure 5 show the dyeing kinetics of two single azo class high molecular weight disperse dyes in the presence of the two auxiliaries used, namely C.I. Disperse Red 167 with 4% o.w.f. of coumarin (Figure 2), C.I. Disperse Red 167 with 4% o.w.f. of *o*-vanillin (Figure 3), Disperse Blue 79 with 4% o.w.f. of coumarin (Figure 4) and Disperse Blue 79 with 4% o.w.f. of *o*-vanillin (Figure 5).

As shown, the initial dyeing rate of coumarin was slightly higher than *o*-vanillin at almost all temperatures, but the final rate of *o*-vanillin was higher than coumarin. Exhaustion % of *o*-vanillin at 100 °C after 120 min was 88.0%, which is similar to that achieved during the conventional dyeing process of polyester at 130–135 °C. Values obtained with *o*-vanillin were slightly higher than coumarin, except for 83 °C, in which dye absorption level was higher.

Concerning the dyeing with Disperse Blue 79, it was found that the initial dyeing rate with coumarin was significantly higher than for *o*-vanillin, except at 100 °C. However, after 60 min of the dyeing process, the PES fabric dyed in presence of *o*-vanillin presented a higher absorption level. At 100 °C, the values of exhaustion of PES fabric dyed with *o*-vanillin reached 87% after 120 min, which is similar to those achieved in the conventional dyeing process of polyester at 130–135 °C, as was mentioned above for Disperse Red 167. 

From these results it can be concluded that *o*-vanillin leads to higher absorption dyeing levels than coumarin. This can be due to the higher hydrophilicity of *o*-vanillin, which makes it more difficult to diffuse it inside of the amorphous phase of PES. However, once diffused, it leads to higher absorption.

Table 3 and Table 4 indicates *K*/*S* values obtained for the dyed polyester fabrics at 100 °C at different times.

As expected, the *K*/*S* values increased as time increased for both auxiliaries at 100 °C. As seen, *o*-vanillin yield higher colour strength than coumarin in all cases. On the other hand, it was found that disperse Red 167 had higher shade depth compared to Disperse Blue 79. Values of *K*/*S* were almost similar to those achieved in the conventional dyeing process of PES at higher temperatures, with a range between 12 and 21.

The following equation to calculate the dyeing rate constant and correlation coefficient was used [11]:

(3) $$K_{T}=\frac{X}{{\left(1-X\right)}^{2}}+0.5\ln\frac{1-X}{1+X}$$

where *x* is the quotient between the dye concentration on the fibre at time *t* and the initial time in the dye-bath, *K* is the dyeing rate constant and t is the time of the kinetics examined.

Table 5 shows the dyeing absorption rate constants and their respective correlation coefficients, calculated from Equation (3).

According to Table 5, the absorption rate constant for both dyes increased with increasing temperature, as expected. The rate constant values of *o*-vanillin were considerably higher than coumarin due to a higher absorption level. Correlation coefficients with the equation used were more than 0.9, demonstrating a good adjustment. In general, values obtained with *o*-vanillin were more attractive.

### 3.3. Activation Energy

The activation energy for each dye during the dyeing process was calculated from the Arrhenius equation:

*K* = *K*_0_*e*^−*E*/*RT*^ (4)

where *K* is the rate constant, *K*_0_ is the pre-exponential factor, *E* is the activation energy, *T* is the absolute temperature and *R* is the constant of ideal gases.

Table 6 shows the apparent activation energies for the dyeing of polyester with disperse dyes at a low temperature by the absorption rate constant obtained from Equation (4).

As can be seen, energies ranged from 37 to 59 kcal·mol^–1^ and were similar to those previously reported for the dyeing of polyester with disperse dyes. These values show that temperature significantly influenced this process. The activation energies of the disperse dyes were higher in the presence of *o*-vanillin than coumarin. This means that dyeing PES with *o*-vanillin requires more energy to diffuse the dyestuff into the fabric than coumarin. Indeed, the higher the activation energy, the higher the barrier to diffusion of the dye molecules into the fabric [11,19]. This can be explained because *o*-vanillin is more hydrophilic than coumarin, and therefore the penetration inside of the dye molecules with this auxiliary is more difficult. However, once *o*-vanillin penetrates, it causes higher swelling of polyester and makes the diffusion of the dye easier. Finally, it was found that the correlation coefficients were all acceptable and had values greater than 0.97.

### 3.4. Washing Fastness

Table 7 shows colour fastness for domestic and commercial laundering for the high molecular weight dyes according to the standard methodology, and using the A2S test indicated in this standard [20].

It can be reported that the colour fastness by washing in this dyeing process was totally acceptable, because there was no adverse influence on fastness properties. Staining of the white polyester fabric was good for all dyes and auxiliaries tested. With respect to the white cotton fabric, values were almost similar to the regular level. Values of both auxiliaries and disperse dyes were quite similar, but *o*-vanillin exhibited slightly better colour fastness by washing than coumarin.

### 3.5. Hot Pressing Fastness

Dry heat was applied to dyed and undyed polyester fabric at three temperatures (110, 150 and 200 °C) under pressure to measure colour changes and colour migration to the white fabric due to the steam. Table 8 and Table 9 indicate discharge hot pressing fastness and degradation hot pressing fastness of dyes with higher molecular weight.

Generally, heat fastness of this dyeing method was positive, and no significant changes can be seen for the properties. Degradation hot pressing fastness of both dyes and auxiliaries at lower temperatures, like 83 and 90 °C, were in the regular level (such as 2–3 or 1–2), whereas discharge hot pressing fastness were in the excellent level, as most values were 5. Polyester fabric dyed in this process had good heat stability based on the degradation results. In terms of transforming colour (migration) to the white fabric (discharge hot fastness), the results were in the regular level.

*O*-vanillin showed remarkably better heat fastness than coumarin, and values obtained with *o*-vanillin were more convincing. In terms of two disperse dyes, the results indicated that C.I. Disperse Blue 79 was better than C.I. Disperse Red 167.

## 4. Conclusions

Based on the kinetics and *K*/*S* analysis, it was found that both auxiliaries at temperatures near to 100 °C led to a good dyeing absorption level in PES fabrics.

Comparing the two auxiliaries studied, it was found that polyester dyed in the presence of *o*-vanillin yielded, in general, a higher absorption level and colour strength than in the presence of coumarin. Moreover, a higher colour strength was found for Disperse Red 167 compared to Disperse Blue 79. It is understandable that temperature would have a significant influence in this dyeing system. The effect of temperature on o-vanillin was more apparent, as dyeing PES with *o*-vanillin involves more energy to penetrate the polyester fabric than coumarin. Ranges of energies were similar to those achieved in the conventional process. In terms of applicability and interaction between fabric and dye molecules, the swelling of polyester dyed with *o*-vanillin auxiliary was higher than coumarin; therefore this factor led the fabric to absorb more colour. According to wash and hot-pressing fastness, the auxiliaries were almost similar to each other. Fastness property results were positive, and there were no significant changes of the fastness properties. However, polyester fabric dyed with *o*-vanillin show slightly better wash and heat fastness than coumarin. 

Additionally, we attempted to dye polyester in a manner as ecologically friendly and economical as possible. The percentages of auxiliaries and dyes were at a minimum level, and this method had several advantages, such as low-cost materials, low energy costs, no toxic chemicals, no deformation of the fabric due to low temperature and the possibility of reusing the waste water as a fertiliser. Therefore, this process can be suggested as an ecological alternative method of dyeing polyester because it avoids phenolic compounds. Moreover, it can be recommended to the industry due to the antimicrobial, antioxidant, biodegradability and anti-mutagenic activities of the carriers involved.

## Figures and Tables

**Table 1:** Chemical structure of dyes and auxiliaries.

Dyes and Chemicals	Chemical Structure
C.I. Disperse Red 167 CAS No. = 61968-52-3/26850-12-4 *M*_W_ = 519.93 g·mol^–1^	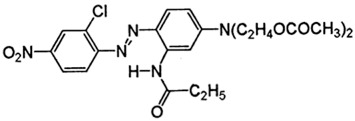
C.I. Disperse blue 79 CAS No. = 12239-34-8 *M*_W_ = 639.41 g·mol^−1^	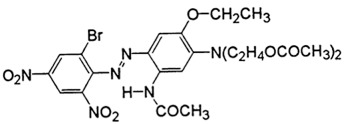
Coumarin 2H-chromen-2-one CAS No. = 91-64-5 *M*_W_ = 146.15 g·mol^−1^	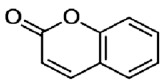
*o*-vanillin 2-hydroxy-3-methoxybenzaldehyde CAS No. = 148-53-8 *M*_W_ = 152.15 g·mol^−1^	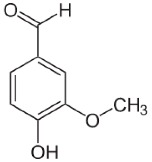

**Table 2:** Particle size of dyebath in a presence of Coumarin, vanillin and without auxiliaries.

Dye-Bath	Average Diameter (nm)	Polydispersity
2% o.w.f. Disperse Red 167; 4% o.w.f. coumarin & 1 mL/g o.w.f. *n*-buthylacetate	183 ± 9.5	153 ± 0.03
2% o.w.f. Disperse Blue 79; 4% o.w.f. coumarin & 1 mL/g o.w.f. *n*-buthylacetate	160.3 ± 14.2	0.121 ± 0.03
2% o.w.f. Disperse Red 167; 4% o.w.f. *o*-vanillin & 1 mL/g o.w.f. *n*-buthylacetate	236.3 ± 10.97	0.107 ± 0.032
2% o.w.f. Disperse Blue 79; 4% o.w.f. *o*-vanillin & 1 mL/g o.w.f. *n*-buthylacetate	249.3 ± 4.5	0.247 ± 0.05
2% o.w.f. Disperse Red 167 & 1 mL/g o.w.f. *n*-buthylacetate	238.3 ± 0.6	0.183 ± 0.01
2% o.w.f. Disperse Blue 79 & 1 mL/g o.w.f. *n*-buthylacetate	250 ± 15.5	0.3 ± 0.05

**Table 3:** *K*/*S* values for PES fabric dyed with D167 in presence of 0.666 g·L^–1^ of coumarin or *o*-vanillin at 100 °C and different times (5, 10, 30, 50, 70, 90 and 105 min).

*T* = 100 °C C.I. Disperse Red 167							
*t* (min)	5	10	30	50	70	90	105
Coumarin							
*K*/*S* λ = 640 nm	4.490	8.820	11.157	16.382	17.025	18.698	19.133
Vanillin							
*K*/*S* λ = 640 nm	3.487	9.260	13.190	14.934	18.378	19.305	21.383

**Table 4:** *K*/*S* values for PES fabric dyed with D79 in presence of 0.666 g·L^–1^ of coumarin or *o*-vanillin at 100 °C and different times (5, 10, 30, 50, 70, 90 and 105 min).

*T* = 100 °C C.I. Disperse Blue 79							
*t* (min)	5	10	30	50	70	90	105
Coumarin							
*K*/*S* λ = 640 nm	3.298	4.807	3.298	4.807	3.298	4.807	3.298
Vanillin							
*K*/*S* λ = 640 nm	3.877	5.061	6.982	7.741	9.982	11.166	12.08

**Table 5:** Absorption rate constant (K), correlation coefficient (R^2^) and exhaustion after 120 min (%) for the kinetics of dyeing of polyester with disperse dyes in the presence of *o*-vanillin or coumarin auxiliaries.

	*o*-Vanillin			Coumarin		
Disperse Dye	*K*	*R*^2^	Exhaustion after 120 min (%)	*K*	*R*^2^	Exhaustion after 120 min (%)
C.I. Disperse Red 167 at 100 °C	0.5667	0.9855	88.21	0.1759	0.9659	81.22
C.I. Disperse Red 167 at 95 °C	0.1712	0.9716	79.78	0.0711	0.9704	71.45
C.I. Disperse Red 167 at 90 °C	0.0426	0.992	65.65	0.0312	0.989	61.96
C.I. Disperse Red 167 at 83 °C	0.0128	0.9523	50.29	0.0153	0.9436	53.45
C.I. Disperse Blue 79 at 100 °C	0.472	0.9764	87.96	0.1892	0.9577	81.42
C.I. Disperse Blue 79 at 95 °C	0.1359	0.9736	78.42	0.0638	0.9268	71.37
C.I. Disperse Blue 79 at 90 °C	0.0722	0.9305	71.95	0.0294	0.934	63.39
C.I. Disperse Blue 79 at 83 °C	0.0208	0.9108	58.34	0.0129	0.9105	53.60413

**Table 6:** Activation energy (E) and correlation coefficient (R^2^) for the two dyes studied in the presence of *o*-vanillin or coumarin auxiliaries.

	*o*-Vanillin		Coumarin	
Disperse Dyes	*E* (Kcal·mol^–1^)	*R*^2^	*E* (Kcal·mol^–1^)	*R*^2^
C.I. Disperse Red 167	59.3	0.9869	37.8	0.9763
C.I. Disperse Blue 79	46.6	0.9841	40.8	0.9781

**Table 7:** Colour fastness by washing of polyester dyed materials.

PET Fabric Dyed 120 min	*o*-Vanillin			Coumarin		
	Gray Scale	PES	Cotton	Gray Scale	PES	Cotton
C.I. Disperse Red 167 at 83 °C	4–5	4	3–4	5	4	3–4
C.I. Disperse Red 167 at 90 °C	5	3–4	3–4	4–5	4–5	3–4
C.I. Disperse Red 167 at 95 °C	5	4–5	3–4	4–5	4	3–4
C.I. Disperse Red 167 at 100 °C	5	4–5	3–4	5	4	3–4
C.I. Disperse Blue 79 at 83 °C	4–5	4–5	3–4	4–5	4–5	3–4
C.I. Disperse Blue 79 at 90 °C	4–5	4–5	3–4	4–5	4–5	3–4
C.I. Disperse Blue 79 at 95 °C	5	5	3–4	5	5	3–4
C.I. Disperse Blue 79 at 100 °C	5	4–5	3	5	4–5	3–4

**Table 8:** Degradation hot pressing fastness.

PET Fabric Dyed 120 min	Discharge Hot Pressing Fastness (Gray Scale)					
	*o*-Vanillin			Coumarin		
	110 °C	150 °C	200 °C	110 °C	150 °C	200 °C
C.I. Disperse Red 167 at 83 °C	5	5	5	5	5	4–5
C.I. Disperse Red 167 at 90 °C	5	5	4–5	5	5	4–5
C.I. Disperse Red 167 at 95 °C	5	5	4–5	5	4–5	4–5
C.I. Disperse Red 167 at 100 °C	5	4–5	4–5	5	4–5	4
C.I. Disperse Blue 79 at 83 °C	5	4–5	4–5	5	5	4–5
C.I. Disperse Blue 79 at 90 °C	5	5	4–5	5	5	4–5
C.I. Disperse Blue 79 at 95 °C	5	4–5	4–5	5	5	4–5
C.I. Disperse Blue 79 at 100 °C	5	5	5	5	5	5

**Table 9:** Discharge Hot Pressing fastness.

PET Fabric Dyed 120 min	Degradation Hot Pressing Fastness (Gray Scale)					
	*o*-Vanillin			Coumarin		
	110 °C	150 °C	200 °C	110 °C	150 °C	200 °C
C.I. Disperse Red 167 at 83 °C	4–5	3	2	3	2–3	1–2
C.I. Disperse Red 167 at 90 °C	4–5	3	1–2	3	2–3	1–2
C.I. Disperse Red 167 at 95 °C	4–5	4	2	2–3	2–3	2–3
C.I. Disperse Red 167 at 100 °C	4–5	4	2	4	4	4–5
C.I. Disperse Blue 79 at 83 °C	5	3	1–2	3–4	3	1–2
C.I. Disperse Blue 79 at 90 °C	4	3–4	1–2	3–4	2–3	2
C.I. Disperse Blue 79 at 90 °C	4–5	4–5	2–3	3	2	3
C.I. Disperse Blue 79 at 100 °C	4–5	4–5	2–3	4–5	4–5	4
